# Satellite Cells in Skeletal Muscle of the Hibernating Dormouse, a Natural Model of Quiescence and Re-Activation: Focus on the Cell Nucleus

**DOI:** 10.3390/cells9041050

**Published:** 2020-04-23

**Authors:** Manuela Malatesta, Manuela Costanzo, Barbara Cisterna, Carlo Zancanaro

**Affiliations:** Anatomy and Histology Section, Department of Neurosciences, Biomedicine and Movement Sciences, University of Verona, Strada Le Grazie, 8 I-37134 Verona, Italy; manuela.malatesta@univr.it (M.M.); manuela.costanzo@univr.it (M.C.); carlo.zancanaro@univr.it (C.Z.)

**Keywords:** Hibernation, electron microscopy, immunocytochemistry

## Abstract

Satellite cells (SCs) participate in skeletal muscle plasticity/regeneration. Activation of SCs implies that nuclear changes underpin a new functional status. In hibernating mammals, periods of reduced metabolic activity alternate with arousals and resumption of bodily functions, thereby leading to repeated cell deactivation and reactivation. In hibernation, muscle fibers are preserved despite long periods of immobilization. The structural and functional characteristics of SC nuclei during hibernation have not been investigated yet. Using ultrastructural and immunocytochemical analysis, we found that the SCs of the hibernating edible dormouse, *Glis glis*, did not show apoptosis or necrosis. Moreover, their nuclei were typical of quiescent cells, showing similar amounts and distributions of heterochromatin, pre-mRNA transcription and processing factors, as well as paired box protein 7 (Pax7) and the myogenic differentiation transcription factor D (MyoD), as in euthermia. However, the finding of accumulated perichromatin granules (i.e., sites of storage/transport of spliced pre-mRNA) in SC nuclei of hibernating dormice suggested slowing down of the nucleus-to-cytoplasm transport. We conclude that during hibernation, SC nuclei maintain similar transcription and splicing activity as in euthermia, indicating an unmodified status during immobilization and hypometabolism. Skeletal muscle preservation during hibernation is presumably not due to SC activation, but rather to the maintenance of some functional activity in myofibers that is able to counteract muscle wasting.

## 1. Introduction

Satellite cells (SCs) represent a population of postnatal mononucleated stem cells [1] that are located between the basal lamina and the sarcolemma of skeletal muscle fibers, and are clearly detectable by means of electron microscopy [2]. SCs are able to occasionally fuse with muscle fibers in order to compensate for the muscle turnover caused by daily wear and tear, or support muscle hypertrophy, thereby underpinning skeletal muscle plasticity [3,4]. SCs exert their physiological role in close interplay with their local environment (the SC niche; [5]). While several non-SC cell populations are involved in muscle plasticity [6], SCs represent a key factor in muscle growth and regeneration, and research thereupon is steadily increasing over time. SCs are typically in a quiescent state, showing a minimum amount of cytoplasm and organelles therein [7]. Upon stimulation (e.g., physical exercise or muscle damage), SCs activate and re-enter the cell cycle, leading to proliferation and/or differentiation. Activation of SCs implies that a transition takes place in the cell nucleus from a low/absent to a high transcriptional activity [8]. The most obvious morphological counterpart of such a transition is a reduction in the amount of heterochromatin, and it was shown that the activation and differentiation of SCs are characterized by an important shift from condensed to lightly packed chromatin [9].

Hibernation is an adaptation to adverse winter conditions adopted by several mammals [10], which is characterized by greatly reduced metabolic activity and lowered body temperature while maintaining homeostasis. Upon arousal, bodily functions are resumed in full. Accordingly, cells in the organs of hibernating animals undergo periodical cycles of deactivation and reactivation. Intriguingly, skeletal muscle mass and strength are preserved during hibernation as well as the fiber size despite long periods of immobilization, contrary to what happens in non-hibernating mammals [11,12,13]. Protective mechanisms apparently take place in skeletal muscle during hibernation, which most likely involve, among others, inhibition of proteolysis, a decrease in autophagy and increased oxidative capacity [14,15]. The preservation of muscle mass during hibernation could also involve SC activation; however, the role of SCs in the prevention of atrophy has barely been investigated [16,17]. In particular, to the best of our knowledge, the structural and functional characteristics of the cell nucleus in SCs during hibernation have not been investigated so far.

Over the last several years, the morphology of the cell nucleus during the hibernation/arousal cycle has been studied in several tissue types of different species in our laboratory [18,19,20,21,22,23,24,25,26,27]. In the skeletal muscle, we found that the fine structure of the muscle fiber is well preserved during hibernation in the edible dormouse (*Glis glis*, Gliridae), with myonuclei showing morphological evidence of transcriptional activity [28]. In the present work, we investigated whether hibernation affects the structural and functional features of the SC nucleus. Both the ultrastructural and immunocytochemical characteristics of the SC nuclei were analyzed in active and hibernating edible dormice, with a focus on the key nuclear constituents involved in RNA transcription and processing. The results show that the SC nuclei are similar in hibernating and euthermic dormice, suggesting that factors other than SC activation are operating during hibernation in the immobilized skeletal muscle to prevent atrophy.

## 2. Materials and Methods

This is a retrospective study conducted on specimens obtained from six male edible dormice captured in 1998/1999 for the purpose of multiple investigations upon permission from the Regione del Veneto (Decreto n. 76 del 20 Gennaio, 1998). Adult (approximately 1–2-year-old) wild-living animals were trapped and maintained in an outdoor animal house supplied with food and bedding material. Under such conditions, they spontaneously began to hibernate in November and awoke in March. Three animals were killed during the euthermic period (June–July), and three during deep hibernation (January, after at least three days of continuous torpor). Euthermic animals were decapitated under deep anesthesia; hibernating animals were taken from the cage and immediately decapitated.

Samples of the right quadriceps muscle were processed for transmission electron microscopy for either morphology or immunocytochemistry.

For morphological analysis, muscle samples were fixed by immersion in 2.5% (*v*/*v*) glutaraldehyde and 2% (*v*/*v*) paraformaldehyde in 0.1 M Sørensen phosphate buffer pH 7.4 at 4 °C for 2 h, post-fixed with 1% (*v*/*v*) OsO_4_ at 4 °C for 1 h, dehydrated through graded acetone and embedded in Epon 812. Ultrathin sections (70–80 nm in thickness) were placed on copper grids coated with a Formvar layer and stained with Reynold’s lead citrate prior to observation.

For immunocytochemistry, muscle samples were fixed by immersion in 4% (*v*/*v*) paraformaldehyde in 0.1 M Sørensen phosphate buffer at 4 °C for 2 h, washed, treated with 0.5 M NH_4_Cl in PBS 0.1 M pH 7.4 to block free aldehydes, dehydrated with ethanol and embedded in LR White resin polymerized under UV light. Ultrathin sections (70–80 nm in thickness) were placed on nickel grids coated with a Formvar-carbon layer and treated with the following probes: mouse monoclonal antibodies directed against the active phosphorylated form of RNA polymerase II (Abcam, Cambridge, MA, USA; ab24759) and the DNA/RNA hybrid molecules [29], both occurring at pre-mRNA transcription sites [28,30]; the (Sm)snRNP (small nuclear RiboNucleoProtein) core protein (Abcam; ab3138), involved in the co-transcriptional splicing of pre-mRNA [31]; the myogenic differentiation transcription factor D (MyoD; [32] (Abcam; ab16148)); and rabbit polyclonal antibody directed against the SC-specific paired box protein 7 (Pax7) transcription factor [33] (Abcam; ab34360). In detail, the sections were floated for 3 min on normal goat serum (NGS) diluted 1:100 in PBS, incubated for 17 h at 4 °C with the primary antibody diluted in PBS containing 0.1% (*w*/*v*) bovine serum albumin (Fluka, Buchs, Switzerland) and 0.05% (*v*/*v*) Tween 20. After rinsing, sections were floated on NGS, and then reacted for 30 min at room temperature with the specific secondary 12 or 6 nm gold-conjugated antibody (Jackson ImmunoResearch Laboratories Inc., West Grove, PA, USA) diluted 1:10 in PBS. Finally, the sections were rinsed and air-dried. The control grids were treated as above, but the primary antibody was omitted from the incubation mixture, and then processed as described. After the immunocytochemical procedure, the sections were treated with Uranyl Acetate Replacement Stain (Electron Microscopy Sciences, Hatfield, PA, USA) for 30 min and lead citrate for 45 s in order to weakly stain the heterochromatin and visualize the structural constituents in the interchromatin space. All grids were observed in a Philips Morgagni TEM operating at 80 kV and equipped with a Megaview III camera for digital image acquisition.

Quantitative assessment of immunolabelling was carried out by estimating the gold particle density over the interchromatin space (i.e., the nucleoplasmic region devoid of heterocromatin clumps) in sections treated in the same run. Briefly, the surface areas of the nucleoplasmic region and the heterochromatin were measured in 10 randomly selected electron micrographs (×22,000) of SC nuclei from each animal using a computerized image analysis system (AnalySIS Image processing, Soft Imaging System GmbH, Münster, Germany). The interchromatin space area was calculated, the gold particles present over the interchromatin space were counted and their density was expressed as number/μm^2^. Background evaluation was carried out on the resin (in the areas devoid of tissue) of the immunolabelled samples as well as on the tissue of control samples. The same procedure was used to assess the density of perichromatin granules (PG; representing sites of storage and/or transport of spliced pre-mRNA [34]) over the interchromatin space.

To estimate the amount of heterochromatin, the percentage of the heterochromatin area within the total nucleoplasm area was calculated.

For each analyzed variable, the Kolmogorov–Smirnov two-sample test was performed in order to verify the hypothesis of identical distribution among animals in each group, and then the mean ± standard error of the mean (SEM) was calculated. Comparisons of variables in the two groups (euthermic and hibernating) were performed with one-way ANOVA (significance set at *p* ≤ 0.05).

## 3. Results

**Figure 1:**
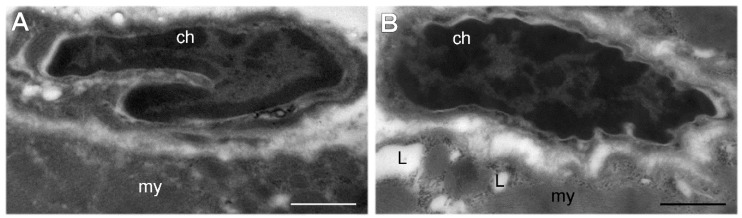
Transmission electron micrographs of satellite cells (SCs) bordering a myofiber (my) in skeletal muscles from euthermic (**A**) and hibernating (**B**) dormice. In both seasonal phases, SC nuclei contain large amounts of heterochromatin (ch). (**B**) The accumulation of lipid droplets (L) in the myofiber is a typical feature of hibernating edible dormice [28]. Bars: 1 μm.

In all of the muscle samples, SCs were morphologically recognizable as small cells located between the sarcolemma and the myofiber basal lamina; they showed scarce cytoplasm and an ovoid nucleus with an irregular border and abundant heterochromatin clumps (Figure 1).

**Figure 2:**
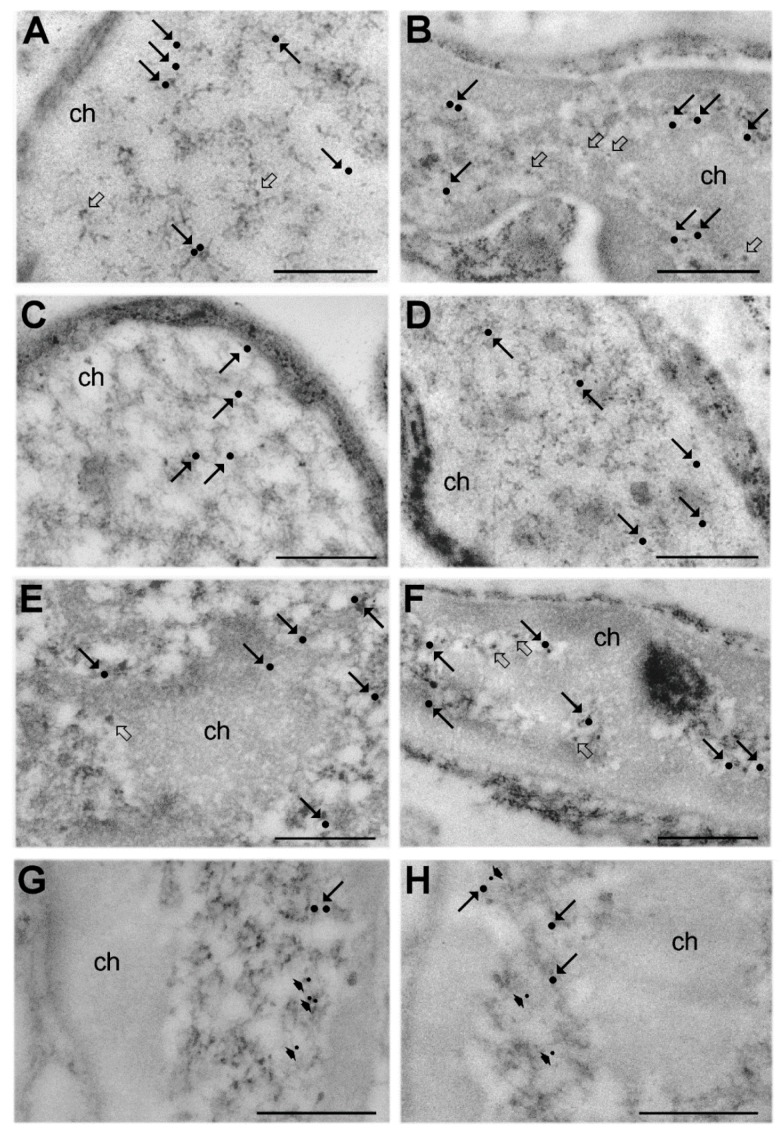
Immunoelectron microscopy. SC nuclei from euthermic (**A**,**C**,**E**,**G**) and hibernating (**B**,**D**,**F**,**H**) dormice; immunolabelling for RNA polymerase II (**A**,**B**; arrows), DNA/RNA hybrid molecules (**C**,**D**; arrows), small nuclear RiboNucleoProtein ((Sm)snRNP) core protein (**E**,**F**; arrows), paired box protein 7 (Pax7) (**G**,**H**; arrows) and the myogenic differentiation transcription factor D (MyoD) (**G**,**H**; arrowheads). All antibodies specifically label perichromatin fibrils (PFs) that mostly occur at the periphery of heterochromatin clumps (ch). Perichromatin granules (PGs) are indicated by open arrows (**A**,**B**,**E**,**F**). Gold particles were digitally enhanced to improve their visibility. Bars: 500 nm.

In the LR White embedded samples, the usual RNP structural constituents involved in pre-mRNA transcription and processing were evident in the nucleoplasm (Figure 2): a few perichromatin fibrils (PFs; representing the in situ form of nascent transcripts, as well as of their splicing and 3′ end processing [34,35]) and PGs were mainly distributed at the periphery of the heterochromatin clumps, and small clusters of interchromatin granules (IG; representing the storage, assembly and phosphorylation sites for transcription and splicing factors [34]) occurred in the interchromatin space (not shown).

**Figure 3:**
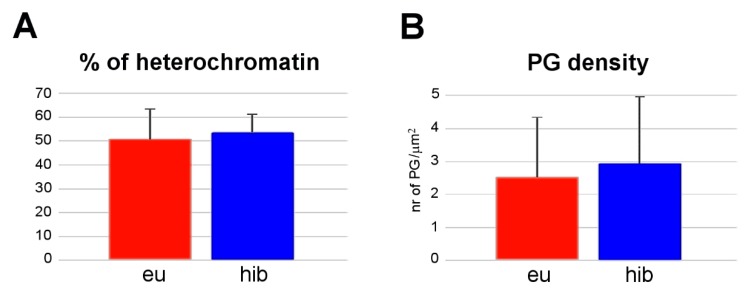
Quantitative evaluation of the percentage of heterochromatin (**A**) and PG density (**B**) (mean ± standard error of the mean (SEM)) in SC nuclei from skeletal muscles of euthermic (eu) and hibernating (hib) dormice. No significant difference was found between euthermia and hibernation for heterochromatin (*p* = 0.091), whereas PG density was significantly higher in hibernating dormice (*p* = 0.002).

SC nuclei were structurally similar in euthermic and hibernating dormice, and morphological evidence of apoptosis or necrosis was never found in any of the muscle samples examined. No statistically significant difference in the percentage of heterochromatin was found between hibernating and euthermic dormice (Figure 3A). Conversely, PG density increased in hibernating vs. euthermic dormice (Figure 3B).

**Figure 4:**
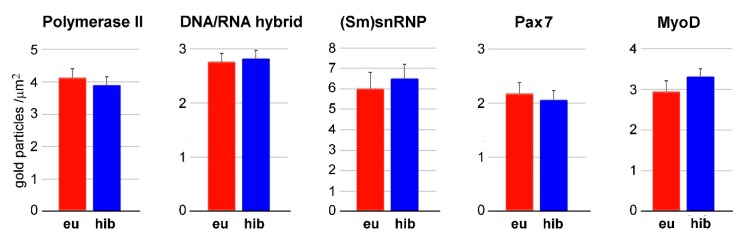
Quantitative immunoelectron microscopy. Labelling density (gold particles/µm^2^) of RNA processing factors in the interchromatin space (mean ± SE) of SC nuclei from skeletal muscles of euthermic (eu) and hibernating (hib) dormice. No significant difference was found between euthermia and hibernation.

The distribution of the immunolabelling for phosphorylated polymerase II, DNA/RNA hybrid molecules, (Sm)snRNP, PAX7 and MyoD was similar in SC nuclei from hibernating and euthermic dormice, being almost exclusively associated with PFs at the edge of the heterochromatin clumps (Figure 2). Quantitative evaluation of the immunolabelling revealed similar densities of all probes in SC nuclei of hibernating and euthermic dormice (Figure 4). Background values were negligible in all the immunolabelling experiments (not shown).

## 4. Discussion

The absence of morphologically recognizable apoptotic or necrotic nuclei in SCs suggests that hibernation does not negatively affect the viability of the SC pool; this is consistent with findings in hindlimb skeletal muscles of late torpid thirteen-lined ground squirrels showing that the number of SCs does not decrease during deep hibernation in comparison with euthermia [16].

The SCs bordering the myofibers of hibernating dormice were morphologically similar to those found in euthermic animals; in particular, the structural features of their nuclei were typical of quiescent cells with low nuclear activity, i.e., showing abundant clumps of heterochromatin and a few PFs [34,35]. Qualitative analysis was confirmed by quantitative evaluation of heterochromatin, which was comparable in euthermic and hibernating dormice (Figure 3), consistent with previous observations in myonuclei of the same hibernating species [28].

Similarly, the in situ analysis of pre-mRNA transcription and processing factors did not reveal differences in both their intranuclear distribution and amount between euthermic and hibernating dormice.

Activated RNA polymerase II and DNA/RNA hybrid molecules as well as snRNPs were specifically located in PFs. Similar results were obtained in myonuclei of the same edible dormice [28] as well as in the liver and brown adipocytes of hazel dormice [20]. This finding suggests that in the SCs of hibernating dormice, the organization of the mRNA transcription and processing machinery is maintained.

Pax7 (a transcription factor marker of both quiescent and active SC) as well as MyoD (a transcription factor increasing in activated SCs [32,33]), were specifically associated with PFs, i.e., sites of transcription. Such an association was previously observed in murine myoblasts in vitro [36], and now, for the first time, in the SCs of the intact muscle.

The presence of similar amounts of immunolabelling for both Pax7 and MyoD in SC nuclei of euthermic and hibernating edible dormice indicates the absence of changes in their activation state along the hibernation cycle, in accordance with findings by Brooks et al. [16] in ground squirrels. Taken together, these results suggest that SC nuclei do not undergo modification in transcription and early splicing during hibernation. However, accumulation of PGs was found during hibernation in these nuclei. Since PGs are involved in storage/transport of spliced pre-mRNA [34], this finding indicates hibernation-associated changes in pre-mRNA processing and/or a slowdown of intranuclear or nucleus-to-cytoplasm transport of mRNAs [37]. Typically, PG accumulation due to the impairment of pre-mRNA processing is accompanied by PF clustering. For example, PF clustering has been found during ageing in different cell types, inclusive of SCs [30,38]. However, in SC nuclei of hibernating edible dormice, no PF clustering was observed. Indeed, an accumulation of PGs unaccompanied by PF clustering was observed in brown adipocytes of hibernating hazel dormice, and was interpreted as a consequence of continuing transcription and splicing activity paralleled by a reduced export of mature mRNA to the cytoplasm, likely to be promptly used upon arousal [39]. Accumulation of PGs during hibernation also suggests that, in spite of the maintenance of transcription and splicing rate, SC activity undergoes some decrease in hibernating edible dormice. This suggestion is supported by findings in hibernating ground squirrels, where an inhibition of both SC activation and myoblast differentiation were shown [17].

Interestingly, no nuclear bodies were observed during hibernation inside the SC nuclei of edible dormice, whereas myonuclei of the same animals showed some amorphous bodies [28]. Different types of nuclear bodies involved in the storage/assembly of RNA processing factors have been shown to form in various tissues during hibernation [23] and rapidly disappear upon arousal when massive nuclear reactivation takes place [21]. Their absence in SCs during hibernation could be related to the naturally quiescent state of these cells, which present a low metabolic activity even in euthermia.

In conclusion, the SC nuclei of hibernating edible dormice maintain similar transcription and splicing activity as in euthermia, although the nucleus-to-cytoplasm transport undergoes a slowing down. Therefore, the SC activation state is unmodified during hibernation, supporting the idea that skeletal muscle preservation during this seasonal phase, characterized by prolonged inactivity and starvation, is not due to SCs, but rather to the maintenance of some functional activity in myofibers that is able to counteract muscle wasting [17,28].
